# Supplementary figures and images for: Pooled testing of traced contacts under superspreading dynamics
Source: PLoS Comput Biol. 2022 Mar 28;18(3):e1010008. doi: 10.1371/journal.pcbi.1010008 (PMC8989305; doi:10.1371/journal.pcbi.1010008)

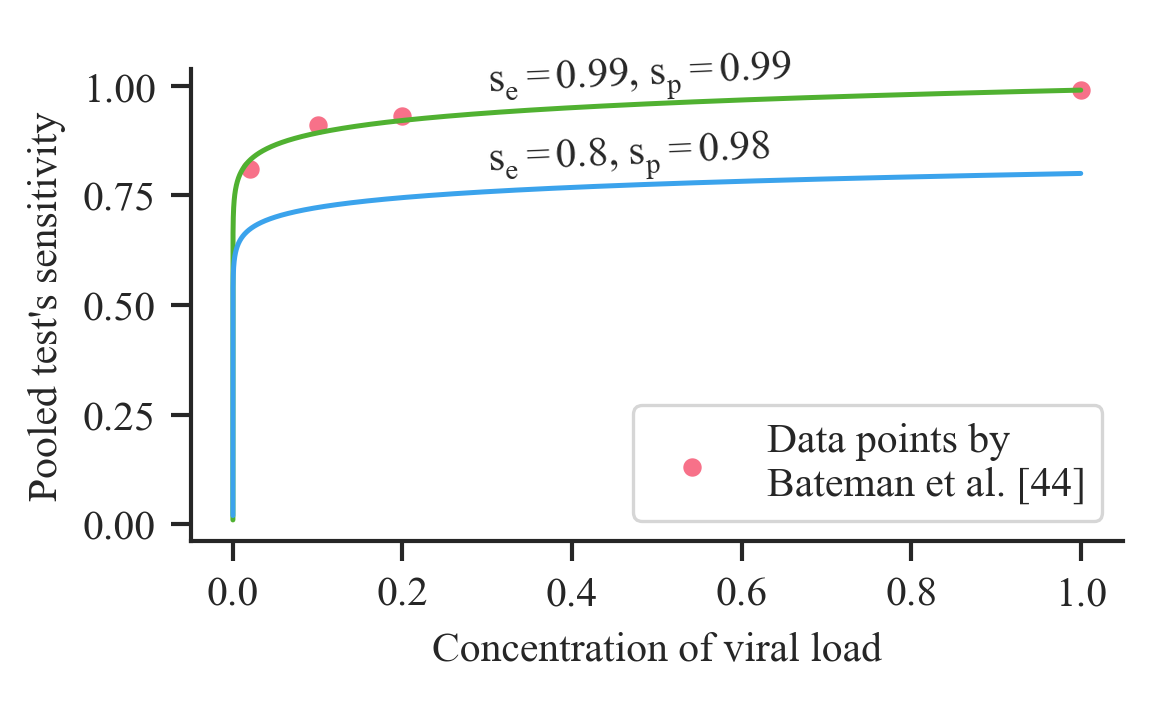

Supplement: S1 Fig — The two lines show the sensitivity of a pooled test as a function of the concentration of viral load based on the parameterized model of P(T(S)=1|I(S)=s>0). The green line shows a pooled test’s analytic sensitivity (high se, sp values) which is fitted based on dilution data by Bateman et al. [44] and gives an estimate of d = 0.0455, via ridge regression. The blue line shows a pooled test’s clinical sensitivity (moderate se and high sp values) under the same value of the dilution parameter d. (TIF) [file pcbi.1010008.s001.tif]

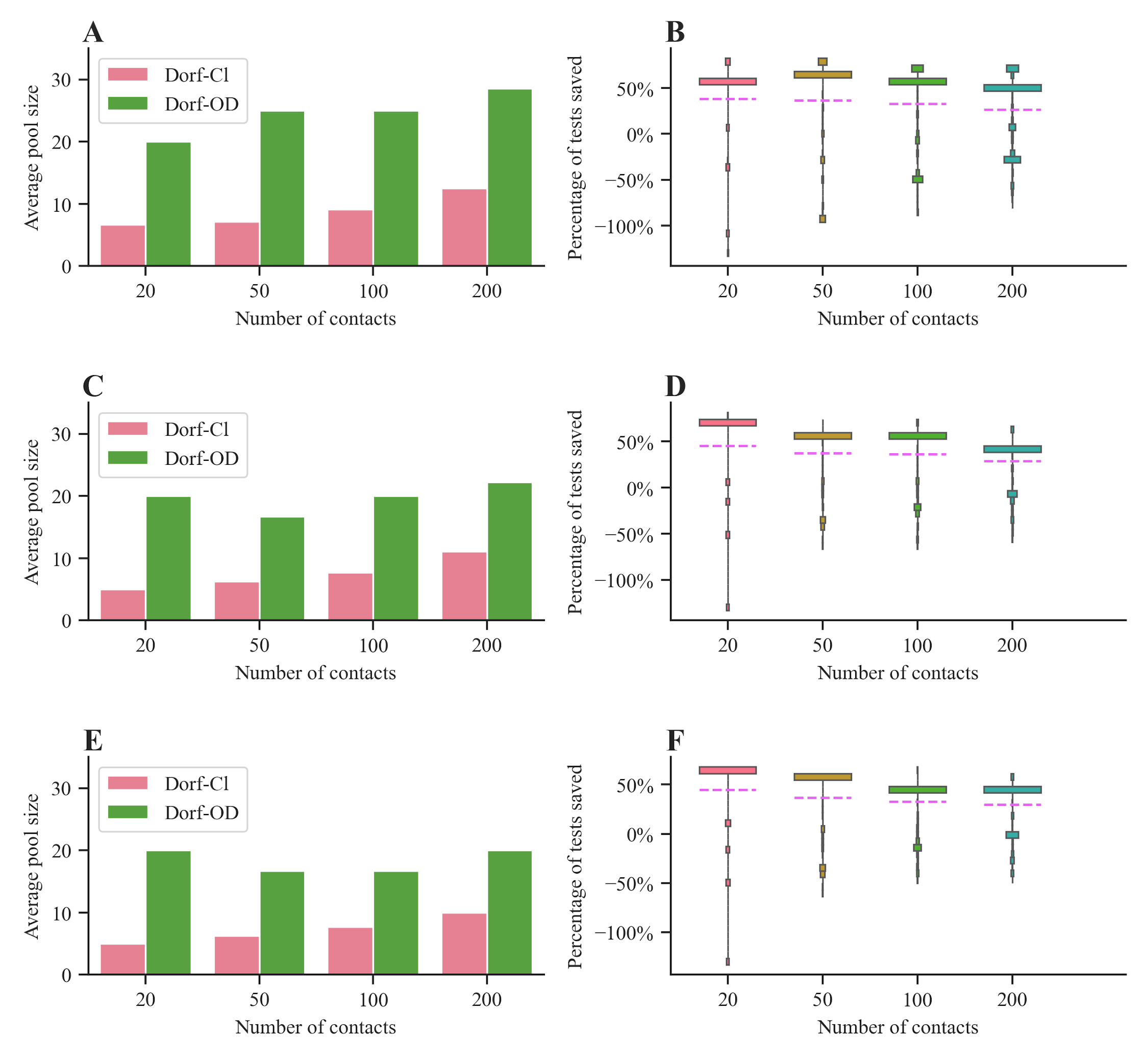

Supplement: S2 Fig — In panels (A, B) we set se = 0.7, sp = 0.97, in panels (C, D) we set se = 0.9, sp = 0.99 and, in panels (E, F) we set se = 0.99, sp = 0.99. Panels (A, C, E) show the average pool size. Panels (B, D, F) show the empirical distribution of the percentage of tests saved by using our method instead of Dorfman’s method, where we exclude the highest and lowest 5% of observations and the purple dashed lines represent average values. In all panels, we sample the number of secondary infections from a truncated negative binomial distribution with reproductive number R = 2.5 and dispersion parameter k = 0.1 [24]. For each combination of method and parameter values, the averages and quantiles in all panels are estimated using 10,000 samples. (TIF) [file pcbi.1010008.s002.tif]

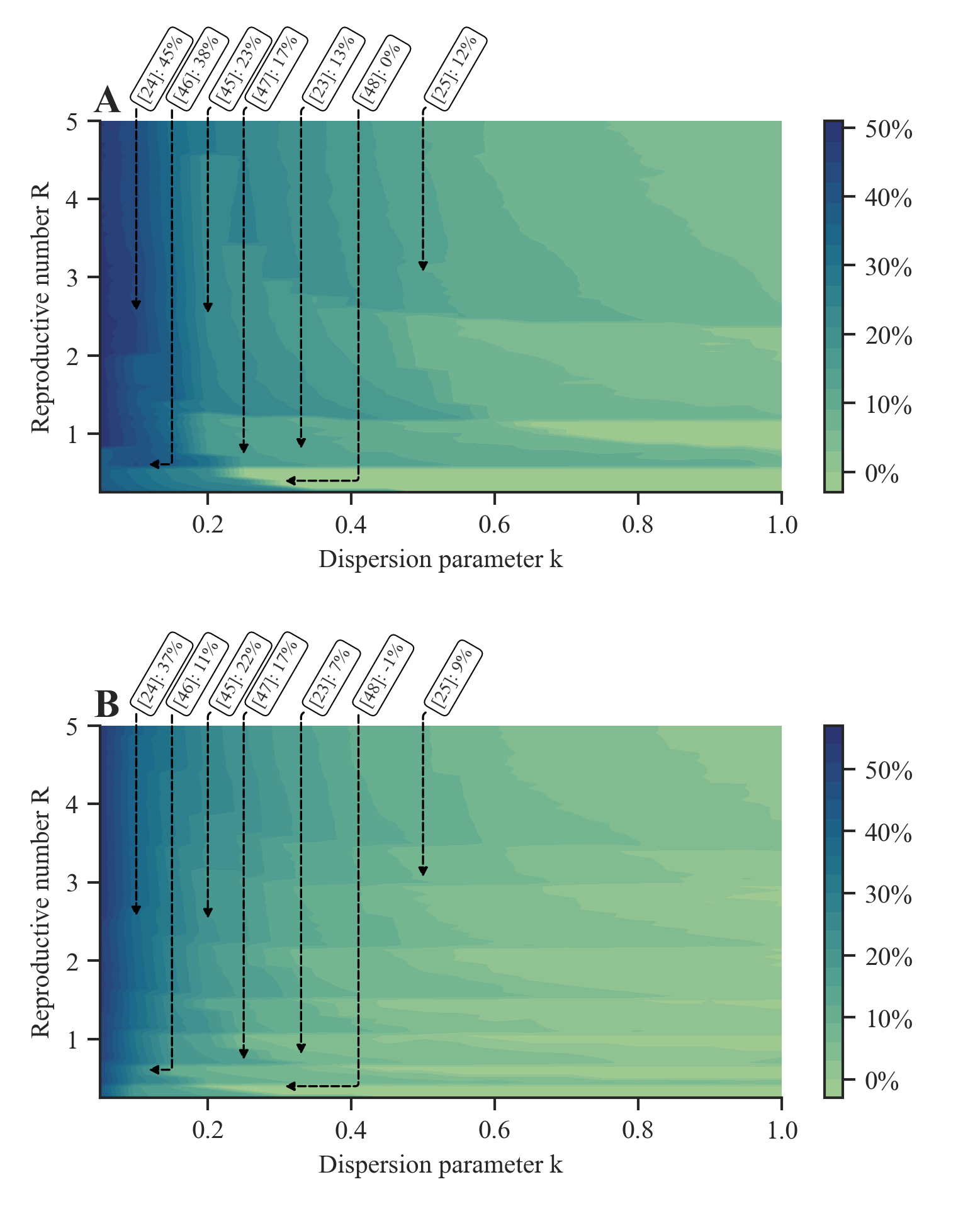

Supplement: S3 Fig — In panels (A, B), we set N = 20 and N = 50 respectively and, in both panels, we set the sensitivity and specificity to se = 0.8, sp = 0.98. Darker colors correspond to a higher average percentage of tests saved. To generate the contours, we evaluate the average percentage of tests saved using values in [0.25, 5.0] with step 0.05 for R and in [0.05, 1.0] with step 0.05 for k. The overlaid annotations indicate the average percentage of tests saved for several estimated values of the reproductive number and dispersion parameter reported in the COVID-19 literature [23–25,45–48]. In each experiment, we estimate the average using 10,000 samples. (TIF) [file pcbi.1010008.s003.tif]

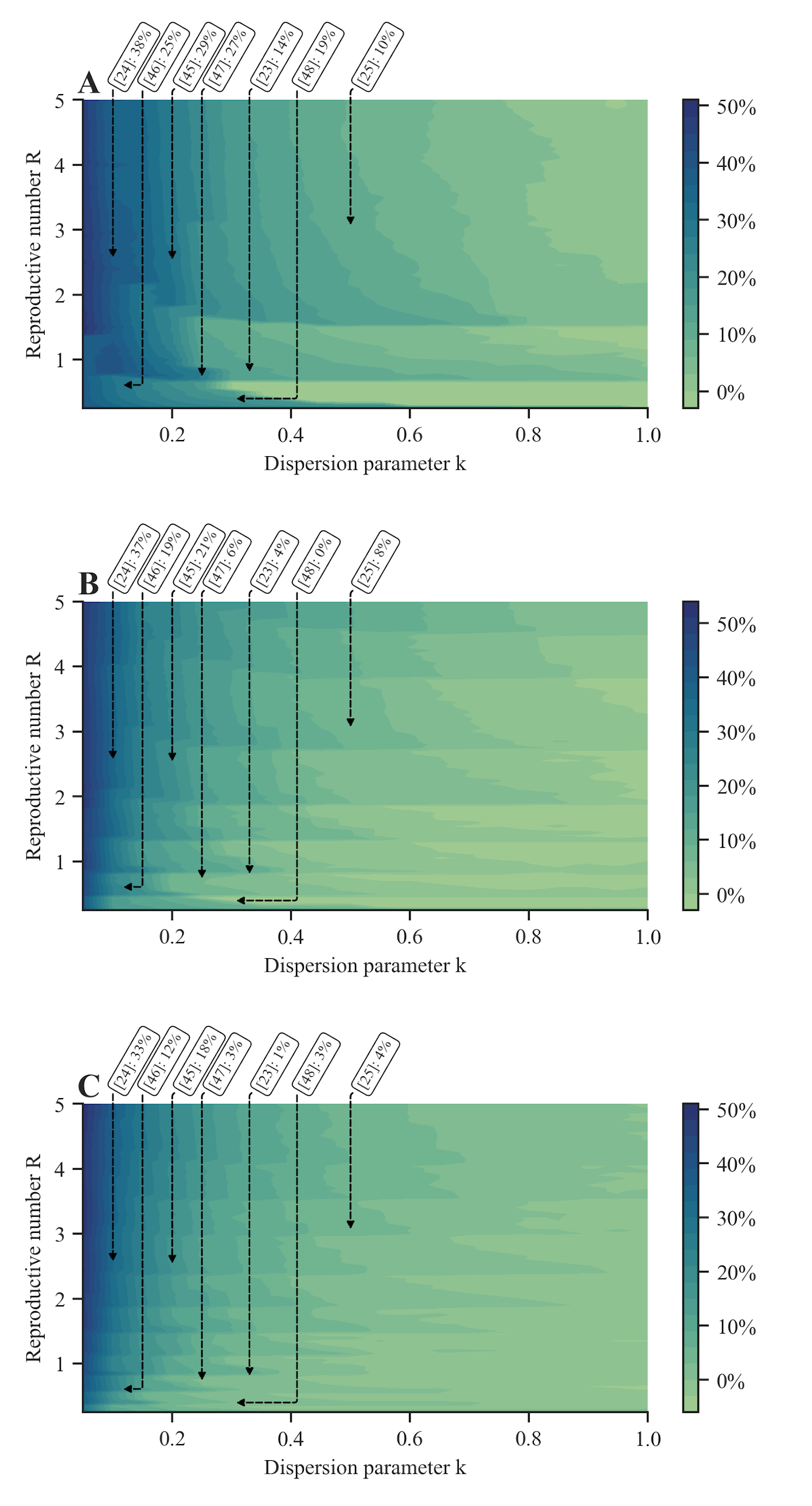

Supplement: S4 Fig — In panels (A, B, C), we set N = 20, N = 50 and N = 100 respectively and, in all panels, we set the sensitivity and specificity to se = 0.7, sp = 0.97. Darker colors correspond to a higher average percentage of tests saved. To generate the contours, we evaluate the average percentage of tests saved using values in [0.25, 5.0] with step 0.05 for R and in [0.05, 1.0] with step 0.05 for k. The overlaid annotations indicate the average percentage of tests saved for several estimated values of the reproductive number and dispersion parameter reported in the COVID-19 literature [23–25,45–48]. In each experiment, we estimate the average using 10,000 samples. (TIF) [file pcbi.1010008.s004.tif]

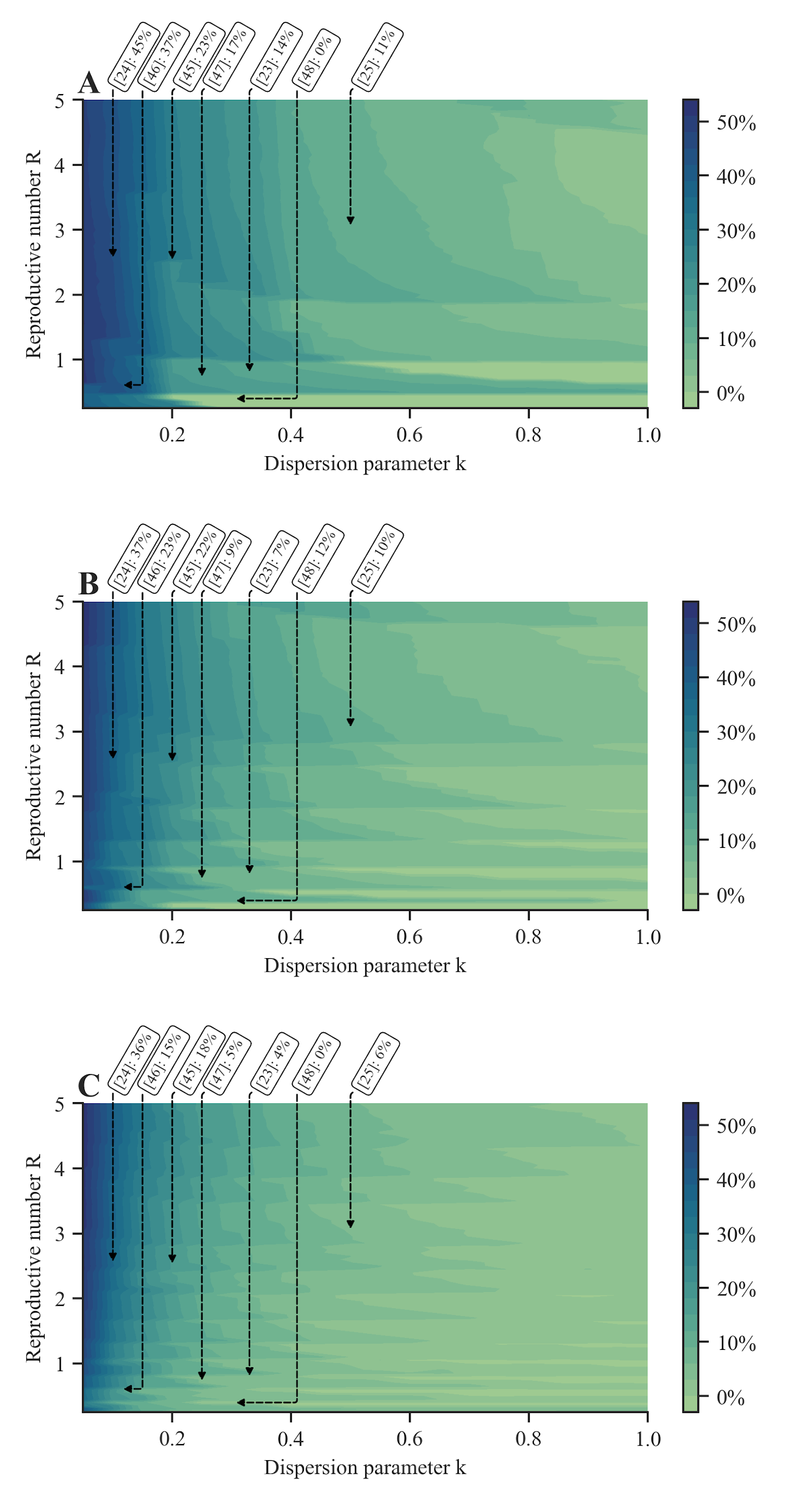

Supplement: S5 Fig — In panels (A, B, C), we set N = 20, N = 50 and N = 100 respectively and, in all panels, we set the sensitivity and specificity to se = 0.9, sp = 0.99. Darker colors correspond to a higher average percentage of tests saved. To generate the contours, we evaluate the average percentage of tests saved using values in [0.25, 5.0] with step 0.05 for R and in [0.05, 1.0] with step 0.05 for k. The overlaid annotations indicate the average percentage of tests saved for several estimated values of the reproductive number and dispersion parameter reported in the COVID-19 literature [23–25,45–48]. In each experiment, we estimate the average using 10,000 samples. (TIF) [file pcbi.1010008.s005.tif]
