# Supplementary material for: Pooled testing of traced contacts under superspreading dynamics
Source: PLoS Comput Biol. 2022 Mar 28;18(3):e1010008. doi: 10.1371/journal.pcbi.1010008 (PMC8989305; doi:10.1371/journal.pcbi.1010008)
Supplement: S1 Table — Here, we set the sensitivity and specificity to se = 0.7, sp = 0.97. We sample the number of secondary infections from a truncated negative binomial distribution with reproductive number R = 2.5 and dispersion parameter k = 0.1 [24] and, for each combination of method and parameter values, the averages and standard deviations are estimated using 10,000 samples. (DOCX) [file pcbi.1010008.s006.docx]

**S1 Table.** **Average numbers of tests, false negatives and false positives of our method (Dorf-OD) and classic Dorfman’s method (Dorf-Cl) for various values of the number of contacts N, under additional levels of sensitivity s_e_ and specificity s_p_.** Here, we set the sensitivity and specificity to **s_e_ = 0.7, s_p_ = 0.97**. We sample the number of secondary infections from a truncated negative binomial distribution with reproductive number R = 2.5 and dispersion parameter k = 0.1 [1] and, for each combination of method and parameter values, the averages and standard deviations are estimated using 10,000 samples.

| N | Average # of tests per contact | | Average # of false negatives per contact | | Average # of false positives per contact | |
| --- | --- | --- | --- | --- | --- | --- |
|  | Dorf-Cl | Dorf-OD | Dorf-Cl | Dorf-OD | Dorf-Cl | Dorf-OD |
| 20 | 0.284  (σ: 0.253) | 0.227  (σ: 0.382) | 0.033  (σ: 0.095) | 0.034  (σ: 0.106) | 0.003  (σ: 0.013) | 0.004  (σ: 0.016) |
| 50 | 0.255  (σ: 0.197) | 0.205  (σ: 0.305) | 0.022  (σ: 0.066) | 0.022  (σ: 0.072) | 0.003  (σ: 0.008) | 0.004  (σ: 0.012) |
| 100 | 0.206  (σ: 0.163) | 0.172  (σ: 0.235) | 0.013  (σ: 0.041) | 0.013  (σ: 0.043) | 0.002  (σ: 0.006) | 0.004  (σ: 0.009) |
| 200 | 0.163  (σ: 0.138) | 0.146  (σ: 0.192) | 0.006  (σ: 0.021) | 0.006  (σ: 0.021) | 0.002  (σ: 0.005) | 0.003  (σ: 0.006) |

**Reference**

1. Endo A, Abbott S, Kucharski AJ, Funk S. Estimating the overdispersion in COVID-19 transmission using outbreak sizes outside China. Wellcome Open Res. 2020;5: 67. doi:10.12688/wellcomeopenres.15842.3
