# Supplementary material for: Pooled testing of traced contacts under superspreading dynamics
Source: PLoS Comput Biol. 2022 Mar 28;18(3):e1010008. doi: 10.1371/journal.pcbi.1010008 (PMC8989305; doi:10.1371/journal.pcbi.1010008)
Supplement: S2 Table — Here, we set the sensitivity and specificity to se = 0.9, sp = 0.99. We sample the number of secondary infections from a truncated negative binomial distribution with reproductive number R = 2.5 and dispersion parameter k = 0.1 [24] and, for each combination of method and parameter values, the averages and standard deviations are estimated using 10,000 samples. (DOCX) [file pcbi.1010008.s007.docx]

**S2 Table.** **Average numbers of tests, false negatives and false positives of our method (Dorf-OD) and classic Dorfman’s method (Dorf-Cl) for various values of the number of contacts N, under additional levels of sensitivity s_e_ and specificity s_p_.** Here, we set the sensitivity and specificity to **s_e_ = 0.9, s_p_ = 0.99**. We sample the number of secondary infections from a truncated negative binomial distribution with reproductive number R = 2.5 and dispersion parameter k = 0.1 [1] and, for each combination of method and parameter values, the averages and standard deviations are estimated using 10,000 samples.

| N | Average # of tests per contact | | Average # of false negatives per contact | | Average # of false positives per contact | |
| --- | --- | --- | --- | --- | --- | --- |
|  | Dorf-Cl | Dorf-OD | Dorf-Cl | Dorf-OD | Dorf-Cl | Dorf-OD |
| 20 | 0.337  (σ: 0.272) | 0.260  (σ: 0.408) | 0.013  (σ: 0.046) | 0.014  (σ: 0.062) | 0.001  (σ: 0.006) | 0.002  (σ: 0.009) |
| 50 | 0.275  (σ: 0.232) | 0.225  (σ: 0.306) | 0.009  (σ: 0.031) | 0.010  (σ: 0.036) | 0.001  (σ: 0.004) | 0.001  (σ: 0.006) |
| 100 | 0.222  (σ: 0.193) | 0.186  (σ: 0.263) | 0.005  (σ: 0.018) | 0.006  (σ: 0.022) | 0.001  (σ: 0.003) | 0.001  (σ: 0.004) |
| 200 | 0.166  (σ: 0.162) | 0.149  (σ: 0.212) | 0.003  (σ: 0.010) | 0.003  (σ: 0.010) | 0.001  (σ: 0.002) | 0.001  (σ: 0.003) |

**Reference**

1. Endo A, Abbott S, Kucharski AJ, Funk S. Estimating the overdispersion in COVID-19 transmission using outbreak sizes outside China. Wellcome Open Res. 2020;5: 67. doi:10.12688/wellcomeopenres.15842.3
