# Supplementary material for: Pooled testing of traced contacts under superspreading dynamics
Source: PLoS Comput Biol. 2022 Mar 28;18(3):e1010008. doi: 10.1371/journal.pcbi.1010008 (PMC8989305; doi:10.1371/journal.pcbi.1010008)
Supplement: S3 Table — Here, we set the sensitivity and specificity to se = 0.99, sp = 0.99. We sample the number of secondary infections from a truncated negative binomial distribution with reproductive number R = 2.5 and dispersion parameter k = 0.1 [24] and, for each combination of method and parameter values, the averages and standard deviations are estimated using 10,000 samples. (DOCX) [file pcbi.1010008.s008.docx]

**S3 Table.** **Average numbers of tests, false negatives and false positives of our method (Dorf-OD) and classic Dorfman’s method (Dorf-Cl) for various values of the number of contacts N, under additional levels of sensitivity s_e_ and specificity s_p_.** Here, we set the sensitivity and specificity to **s_e_ = 0.99, s_p_ = 0.99**. We sample the number of secondary infections from a truncated negative binomial distribution with reproductive number R = 2.5 and dispersion parameter k = 0.1 [1] and, for each combination of method and parameter values, the averages and standard deviations are estimated using 10,000 samples.

| N | Average # of tests per contact | | Average # of false negatives per contact | | Average # of false positives per contact | |
| --- | --- | --- | --- | --- | --- | --- |
|  | Dorf-Cl | Dorf-OD | Dorf-Cl | Dorf-OD | Dorf-Cl | Dorf-OD |
| 20 | 0.350  (σ: 0.292) | 0.282  (σ: 0.422) | 0.003  (σ: 0.018) | 0.004  (σ: 0.030) | 0.001  (σ: 0.007) | 0.002  (σ: 0.010) |
| 50 | 0.286  (σ: 0.252) | 0.242  (σ: 0.330) | 0.003  (σ: 0.011) | 0.003  (σ: 0.016) | 0.001  (σ: 0.004) | 0.001  (σ: 0.006) |
| 100 | 0.230  (σ: 0.211) | 0.199  (σ: 0.271) | 0.002  (σ: 0.007) | 0.002  (σ: 0.009) | 0.001  (σ: 0.003) | 0.001  (σ: 0.004) |
| 200 | 0.179  (σ: 0.170) | 0.159  (σ: 0.223) | 0.001  (σ: 0.004) | 0.001  (σ: 0.005) | 0.001  (σ: 0.002) | 0.001  (σ: 0.003) |

**Reference**

1. Endo A, Abbott S, Kucharski AJ, Funk S. Estimating the overdispersion in COVID-19 transmission using outbreak sizes outside China. Wellcome Open Res. 2020;5: 67. doi:10.12688/wellcomeopenres.15842.3
