# Supplementary material for: Pooled testing of traced contacts under superspreading dynamics
Source: PLoS Comput Biol. 2022 Mar 28;18(3):e1010008. doi: 10.1371/journal.pcbi.1010008 (PMC8989305; doi:10.1371/journal.pcbi.1010008)
Supplement: S4 Table — Here, under se = 0.7, sp = 0.97, we vary λ1 while we fix λ2 = 0 and, for each resulting partition, we compute the average number of tests and false negative/positive rate. We set the number of contacts to N = 100 and sample the number of positive infections from a truncated negative binomial distribution with reproductive number R = 2.5 and dispersion parameter k = 0.1. In each experiment, we estimate averages using 10,000 samples. Double entries in the first column correspond to cases where the set of contacts is partitioned into a combination of pools of two different sizes. (DOCX) [file pcbi.1010008.s009.docx]

S4 Table. Pool partitions corresponding to the points of Fig 3A, resulting by penalizing the false negative rate. Here, under se=0.7, sp=0.97, we vary λ_1_ while we fix λ_2_=0 and, for each resulting partition, we compute the average number of tests and false negative/positive rate. We set the number of contacts to N = 100 and sample the number of positive infections from a truncated negative binomial distribution with reproductive number R = 2.5 and dispersion parameter k = 0.1. In each experiment, we estimate averages using 10,000 samples. Double entries in the first column correspond to cases where the set of contacts is partitioned into a combination of pools of two different sizes.

| Pool partitions  (# of pools x size) | Average # of tests | False Negative Rate | False Positive Rate |
| --- | --- | --- | --- |
| 4 x 25 | 17.20 | 15.15% | 0.40% |
| 5 x 20 | 17.38 | 15.04% | 0.36% |
| 5 x 19  5 x 1 | 21.67 | 14.79% | 0.49% |
| 100 x 1 | 100.00 | 8.16% | 3.00% |
