# Supplementary material for: Pooled testing of traced contacts under superspreading dynamics
Source: PLoS Comput Biol. 2022 Mar 28;18(3):e1010008. doi: 10.1371/journal.pcbi.1010008 (PMC8989305; doi:10.1371/journal.pcbi.1010008)
Supplement: S5 Table — Here, under se = 0.7, sp = 0.97, we vary λ2 while we fix λ1 = 0 and, for each resulting partition, we compute the average number of tests and false negative/positive rate. We set the number of contacts to N = 100 and sample the number of positive infections from a truncated negative binomial distribution with reproductive number R = 2.5 and dispersion parameter k = 0.1. In each experiment, we estimate averages using 10,000 samples. Double entries in the first column correspond to cases where the set of contacts is partitioned into a combination of pools of two different sizes. (DOCX) [file pcbi.1010008.s010.docx]

**S5 Table. Pool partitions corresponding to the points of Fig 3A, resulting by penalizing the false positive rate.** Here, under **se=0.7, sp=0.97**, we **vary λ_2_** while we fix λ_1_=0 and, for each resulting partition, we compute the average number of tests and false negative/positive rate. We set the number of contacts to N = 100 and sample the number of positive infections from a truncated negative binomial distribution with reproductive number R = 2.5 and dispersion parameter k = 0.1. In each experiment, we estimate averages using 10,000 samples. Double entries in the first column correspond to cases where the set of contacts is partitioned into a combination of pools of two different sizes.

| Pool partitions  (# of pools x size) | Average # of tests | False Negative Rate | False Positive Rate |
| --- | --- | --- | --- |
| 4 x 25 | 17.20 | 15.15% | 0.40% |
| 5 x 20 | 17.38 | 15.04% | 0.36% |
| 4 x 17  2 x 16 | 17.66 | 15.07% | 0.35% |
| 2 x 15  5 x 14 | 18.10 | 14.93% | 0.33% |
| 4 x 13  4 x 12 | 18.69 | 14.83% | 0.31% |
| 1 x 12  8 x 11 | 19.25 | 14.67% | 0.30% |
| 10 x 10 | 19.98 | 14.73% | 0.29% |
| 1 x 10  10 x 9 | 20.58 | 14.82% | 0.28% |
| 4 x 9  8 x 8 | 21.34 | 14.54% | 0.28% |
| 9 x 8  4 x 7 | 22.17 | 14.69% | 0.27% |
| 2 x 8  12 x 7 | 22.92 | 14.68% | 0.26% |
| 10 x 7  5 x 6 | 23.65 | 14.69% | 0.25% |
| 4 x 7  12 x 6 | 24.49 | 14.43% | 0.24% |
| 15 x 6  2 x 5 | 25.32 | 14.41% | 0.24% |
| 20 x 5 | 27.84 | 14.58% | 0.22% |
| 25 x 4 | 32.20 | 14.66% | 0.20% |
| 1 x 4  32 x 3 | 39.55 | 14.21% | 0.17% |
| 32 x 3  2 x 2 | 40.46 | 14.23% | 0.17% |
| 50 x 2 | 55.64 | 13.84% | 0.13% |
