# Supplementary material for: Pooled testing of traced contacts under superspreading dynamics
Source: PLoS Comput Biol. 2022 Mar 28;18(3):e1010008. doi: 10.1371/journal.pcbi.1010008 (PMC8989305; doi:10.1371/journal.pcbi.1010008)
Supplement: S6 Table — Here, under se = 0.8, sp = 0.98, we vary λ1 while we fix λ2 = 0 and, for each resulting partition, we compute the average number of tests and false negative/positive rate. We set the number of contacts to N = 100 and sample the number of positive infections from a truncated negative binomial distribution with reproductive number R = 2.5 and dispersion parameter k = 0.1. In each experiment, we estimate averages using 10,000 samples. Double entries in the first column correspond to cases where the set of contacts is partitioned into a combination of pools of two different sizes. (DOCX) [file pcbi.1010008.s011.docx]

**S6 Table. Pool partitions corresponding to the points of Fig 3B, resulting by penalizing the false negative rate.** Here, under **se=0.8, sp=0.98**, we **vary λ_1_** while we fix λ_2_=0 and, for each resulting partition, we compute the average number of tests and false negative/positive rate. We set the number of contacts to N = 100 and sample the number of positive infections from a truncated negative binomial distribution with reproductive number R = 2.5 and dispersion parameter k = 0.1. In each experiment, we estimate averages using 10,000 samples. Double entries in the first column correspond to cases where the set of contacts is partitioned into a combination of pools of two different sizes.

| Pool partitions  (# of pools x size) | Average # of tests | False Negative Rate | False Positive Rate |
| --- | --- | --- | --- |
| 5 x 20 | 17.99 | 11.40% | 0.25% |
| 4 x 17  2 x 16 | 18.15 | 11.52% | 0.24% |
| 2 x 15  5 x 14 | 18.48 | 11.49% | 0.22% |
| 7 x 14  2 x 1 | 20.35 | 11.02% | 0.26% |
| 100 x 1 | 100.00 | 5.43% | 2.00% |
