# Supplementary material for: Pooled testing of traced contacts under superspreading dynamics
Source: PLoS Comput Biol. 2022 Mar 28;18(3):e1010008. doi: 10.1371/journal.pcbi.1010008 (PMC8989305; doi:10.1371/journal.pcbi.1010008)
Supplement: S7 Table — Here, under se = 0.8, sp = 0.98, we vary λ2 while we fix λ1 = 0 and, for each resulting partition, we compute the average number of tests and false negative/positive rate. We set the number of contacts to N = 100 and sample the number of positive infections from a truncated negative binomial distribution with reproductive number R = 2.5 and dispersion parameter k = 0.1. In each experiment, we estimate averages using 10,000 samples. Double entries in the first column correspond to cases where the set of contacts is partitioned into a combination of pools of two different sizes. (DOCX) [file pcbi.1010008.s012.docx]

**S7 Table. Pool partitions corresponding to the points of Fig 3B, resulting by penalizing the false positive rate.** Here, under **se=0.8, sp=0.98**, we **vary λ_2_** while we fix λ_1_=0 and, for each resulting partition, we compute the average number of tests and false negative/positive rate. We set the number of contacts to N = 100 and sample the number of positive infections from a truncated negative binomial distribution with reproductive number R = 2.5 and dispersion parameter k = 0.1. In each experiment, we estimate averages using 10,000 samples. Double entries in the first column correspond to cases where the set of contacts is partitioned into a combination of pools of two different sizes.

| Pool partitions  (# of pools x size) | Average # of tests | False Negative Rate | False Positive Rate |
| --- | --- | --- | --- |
| 5 x 20 | 17.99 | 11.40% | 0.25% |
| 4 x 17  2 x 16 | 18.15 | 11.52% | 0.24% |
| 2 x 15  5 x 14 | 18.47 | 11.49% | 0.22% |
| 4 x 13  4 x 12 | 19.07 | 11.18% | 0.21% |
| 1 x 12  8 x 11 | 19.57 | 11.05% | 0.21% |
| 10 x 10 | 20.20 | 10.97% | 0.19% |
| 1 x 10  10 x 9 | 20.74 | 11.15% | 0.19% |
| 4 x 9  8 x 8 | 21.44 | 10.83% | 0.18% |
| 9 x 8  4 x 7 | 22.22 | 10.94% | 0.18% |
| 2 x 8  12 x 7 | 22.90 | 11.02% | 0.17% |
| 10 x 7  5 x 6 | 23.65 | 11.05% | 0.16% |
| 4 x 7  12 x 6 | 24.40 | 10.84% | 0.16% |
| 15 x 6  2 x 5 | 25.20 | 10.89% | 0.15% |
| 20 x 5 | 27.68 | 10.83% | 0.13% |
| 25 x 4 | 31.91 | 10.74% | 0.12% |
| 1 x 4  32 x 3 | 39.15 | 10.27% | 0.11% |
| 32 x 3  2 x 2 | 40.04 | 10.24% | 0.10% |
| 50 x 2 | 55.09 | 10.00% | 0.08% |
