# Supplementary material for: Pooled testing of traced contacts under superspreading dynamics
Source: PLoS Comput Biol. 2022 Mar 28;18(3):e1010008. doi: 10.1371/journal.pcbi.1010008 (PMC8989305; doi:10.1371/journal.pcbi.1010008)
Supplement: S8 Table — Here, under se = 0.9, sp = 0.99, we vary λ1 while we fix λ2 = 0 and, for each resulting partition, we compute the average number of tests and false negative/positive rate. We set the number of contacts to N = 100 and sample the number of positive infections from a truncated negative binomial distribution with reproductive number R = 2.5 and dispersion parameter k = 0.1. In each experiment, we estimate averages using 10,000 samples. Double entries in the first column correspond to cases where the set of contacts is partitioned into a combination of pools of two different sizes. (DOCX) [file pcbi.1010008.s013.docx]

**S8 Table. Pool partitions corresponding to the points of Fig 3C, resulting by penalizing the false negative rate.** Here, under **se=0.9, sp=0.99**, we **vary λ_1_** while we fix λ_2_=0 and, for each resulting partition, we compute the average number of tests and false negative/positive rate. We set the number of contacts to N = 100 and sample the number of positive infections from a truncated negative binomial distribution with reproductive number R = 2.5 and dispersion parameter k = 0.1. In each experiment, we estimate averages using 10,000 samples. Double entries in the first column correspond to cases where the set of contacts is partitioned into a combination of pools of two different sizes.

| Pool partitions  (# of pools x size) | Average # of tests | False Negative Rate | False Positive Rate |
| --- | --- | --- | --- |
| 5 x 20 | 18.62 | 7.31% | 0.13% |
| 4 x 17  2 x 16 | 18.62 | 7.53% | 0.12% |
| 2 x 15  5 x 14 | 18.90 | 7.31% | 0.11% |
| 4 x 13  4 x 12 | 19.35 | 6.90% | 0.10% |
| 1 x 12  8 x 11 | 19.79 | 6.87% | 0.10% |
| 10 x 10 | 20.34 | 7.02% | 0.09% |
| 1 x 10  10 x 9 | 20.84 | 7.18% | 0.10% |
| 4 x 9  8 x 8 | 21.49 | 6.92% | 0.09% |
| 12 x 8  4 x1 | 25.07 | 6.52% | 0.12% |
| 100 x 1 | 100.00 | 2.77% | 1.00% |
