# Supplementary material for: Pooled testing of traced contacts under superspreading dynamics
Source: PLoS Comput Biol. 2022 Mar 28;18(3):e1010008. doi: 10.1371/journal.pcbi.1010008 (PMC8989305; doi:10.1371/journal.pcbi.1010008)
Supplement: S10 Table — Here, under se = 0.99, sp = 0.99, we vary λ1 while we fix λ2 = 0 and, for each resulting partition, we compute the average number of tests and false negative/positive rate. We set the number of contacts to N = 100 and sample the number of positive infections from a truncated negative binomial distribution with reproductive number R = 2.5 and dispersion parameter k = 0.1. In each experiment, we estimate averages using 10,000 samples. Double entries in the first column correspond to cases where the set of contacts is partitioned into a combination of pools of two different sizes. (DOCX) [file pcbi.1010008.s015.docx]

**S10 Table. Pool partitions corresponding to the points of Fig 3D, resulting by penalizing the false negative rate.** Here, under **se=0.99, sp=0.99**, we **vary λ_1_** while we fix λ_2_=0 and, for each resulting partition, we compute the average number of tests and false negative/positive rate. We set the number of contacts to N = 100 and sample the number of positive infections from a truncated negative binomial distribution with reproductive number R = 2.5 and dispersion parameter k = 0.1. In each experiment, we estimate averages using 10,000 samples. Double entries in the first column correspond to cases where the set of contacts is partitioned into a combination of pools of two different sizes.

| Pool partitions  (# of pools x size) | Average # of tests | False Negative Rate | False Positive Rate |
| --- | --- | --- | --- |
| 4 x 17  2 x 16 | 19.91 | 3.23% | 0.14% |
| 2 x 15  5 x 14 | 20.08 | 3.15% | 0.13% |
| 4 x 13  4 x 12 | 20.43 | 2.92% | 0.12% |
| 1 x 12  8 x 11 | 20.82 | 2.86% | 0.12% |
| 10 x 10 | 21.32 | 2.77% | 0.10% |
| 1 x 10  10 x 9 | 21.77 | 2.85% | 0.10% |
| 4 x 9  8 x 8 | 22.37 | 2.81% | 0.10% |
| 9 x 8  4 x 7 | 23.01 | 2.64% | 0.09% |
| 2 x 8  12 x 7 | 23.66 | 2.69% | 0.09% |
| 10 x 7  5 x 6 | 24.36 | 2.56% | 0.09% |
| 4 x 7  12 x 6 | 25.04 | 2.60% | 0.09% |
| 15 x 6  2 x 5 | 25.82 | 2.38% | 0.08% |
| 20 x 5 | 28.13 | 2.21% | 0.07% |
| 25 x 4 | 32.21 | 1.94% | 0.06% |
| 1 x 4  32 x 3 | 39.21 | 1.70% | 0.05% |
| 32 x 3  2 x 2 | 40.11 | 1.69% | 0.05% |
| 50 x 2 | 54.89 | 1.30% | 0.03% |
| 100 x 1 | 100.00 | 0.30% | 1.00% |
