# Supplementary material for: Pooled testing of traced contacts under superspreading dynamics
Source: PLoS Comput Biol. 2022 Mar 28;18(3):e1010008. doi: 10.1371/journal.pcbi.1010008 (PMC8989305; doi:10.1371/journal.pcbi.1010008)
Supplement: S1 Appendix — (DOCX) [file pcbi.1010008.s017.docx]

**S1 Appendix. Derivations for Dorfman testing under overdispersion (Dorf-OD)**

**Expected number of tests**

Let $K(\mathcal{S)}$ be the number of tests performed when testing pool $\mathcal{S}$ using Dorfman testing. Then, the expected number of tests $\mathbb{E[}K\left( \mathcal{S} \right)]$ due to a pool $\mathcal{S}$ is:

$$\mathbb{E}\left[ K\left( \mathcal{S} \right) \right]=\left\{ \begin{aligned} 1+f\left( \mathcal{S} \right) \left| \mathcal{S} \right|>1 \\ 1 \left| \mathcal{S} \right|=1 \end{aligned} \right. ,$$

where $f(\mathcal{S)}$ is given by

$$f\left( \mathcal{S} \right)=\left| \mathcal{S} \right|\left[ 1-P\left( T\left( \mathcal{S} \right)=0 \right) \right]$$

$$=\left| \mathcal{S} \right|\left[ 1-\sum_{s=0}^{\left| \mathcal{S} \right|} P\left( T\left( \mathcal{S} \right)=0 \right| I\left( \mathcal{S} \right)=s)P(I\left( \mathcal{S} \right)=s) \right]$$

$$=\left| \mathcal{S} \right|\left[ 1-\sum_{s=1}^{\left| \mathcal{S} \right|} P\left( T\left( \mathcal{S} \right)=0 \right| I\left( \mathcal{S} \right)=s)\sum_{n=s}^{N} P\left( I\left( \mathcal{S} \right)=s \right| I\left( \mathcal{N} \right)=n)P(I\left( \mathcal{N} \right)=n)-P\left( T\left( \mathcal{S} \right)=0 \right| I\left( \mathcal{S} \right)=0)\sum_{n=0}^{N} P\left( I\left( \mathcal{S} \right)=0 \right| I\left( \mathcal{N} \right)=n)P\left( I\left( \mathcal{N} \right)=n \right) \right]$$

$$=\left| \mathcal{S} \right|\left[ 1-\sum_{s=1}^{\left| \mathcal{S} \right|} \left( s_{p}- \left( s_{e}+s_{p}-1 \right)\left( \frac{s}{\left| \mathcal{S} \right|} \right)^{d} \right)\sum_{n=s}^{N} \frac{\binom{n}{s}\binom{N-n}{\left| \mathcal{S} \right|-s}}{\binom{N}{\left| \mathcal{S} \right|}}q_{R,k,N}\left( n \right)-s_{p}\sum_{n=0}^{N} \frac{\binom{N-n}{\left| \mathcal{S} \right|}}{\binom{N}{\left| \mathcal{S} \right|}}q_{R,k,N}\left( n \right) \right],$$

where the last step follows from the fact that $I\left( \mathcal{S} \right) | I\left( \mathcal{N} \right)=n \sim HGeom(N, n, \left| \mathcal{S} \right|)$, our assumption about $P(I\left( \mathcal{N} \right)=n)$ and our assumptions about $T(\mathcal{S)}$.

**Expected number of false negatives**

To compute the number of false negatives, we distinguish between two cases. If $\left| \mathcal{S} \right|=1$, i.e., the pool consists of only one person, there is no distinction between a pooled test and an individual test. Therefore, a false negative can occur only if the person is infected and the test turns out negative. Thus,

$$\mathbb{E}\left[ FN\left( \mathcal{S} \right) \right]=\left( 1-s_{e} \right)P\left( I\left( \mathcal{S} \right)=1 \right)=\left( 1-s_{e} \right)\sum_{n=1}^{N} \frac{n}{N}q_{R,k,N}\left( n \right).$$

If $\left| \mathcal{S} \right|>1$, a pooled test is performed and, if it turns out positive, individual tests are performed subsequently. Then, the expected number of false negatives is

$$\mathbb{E}\left[ FN\left( s \right) \right]=\sum_{s=1}^{\mathcal{|S|}} sP\left( T\left( \mathcal{S} \right)=0 \right| I\left( \mathcal{S} \right)=s)P\left( I\left( \mathcal{S} \right)=s \right)+\sum_{s=1}^{\mathcal{|S|}} P\left( T\left( \mathcal{S} \right)=1 \right| I\left( \mathcal{S} \right)=s)P\left( I\left( \mathcal{S} \right)=s \right)s(1-s_{e}),$$

where the first term corresponds to the case where the pooled test outcome is falsely negative and the second term corresponds to the case where the pooled test outcome is truly positive and the individual tests are falsely negative. Finally, using our assumptions about the individual probabilities, we rewrite the above expression as

$$\mathbb{E}\left[ FN\left( s \right) \right]=\sum_{s=1}^{\left| \mathcal{S} \right|} s\left( s_{p}- \left( s_{e}+s_{p}-1 \right)\left( \frac{s}{\left| \mathcal{S} \right|} \right)^{d} \right)P\left( I\left( \mathcal{S} \right)=s \right)+\sum_{s=1}^{\left| \mathcal{S} \right|} s\left( 1-s_{e} \right)\left( 1-s_{p}+\left( s_{e}+s_{p}-1 \right)\left( \frac{s}{\left| \mathcal{S} \right|} \right)^{d} \right)P\left( I\left( \mathcal{S} \right)=s \right)$$

$$=\sum_{s=1}^{\mathcal{|S|}} s\left[ 1-s_{e}+s_{e}\left( s_{p}- \left( s_{e}+s_{p}-1 \right)\left( \frac{s}{\left| \mathcal{S} \right|} \right)^{d} \right) \right]\left[ \sum_{n=s}^{N} \frac{\binom{n}{s}\binom{N-n}{\left| \mathcal{S} \right|-s}}{\binom{N}{\left| \mathcal{S} \right|}}q_{R,k,N}(n) \right].$$

**Expected number of false positives**

Similarly, as with the expected number of false negatives, we distinguish between the two cases. If $\left| \mathcal{S} \right|=1$, there is no distinction between a pooled test and an individual test. Therefore, a false positive can occur only if the person is not infected and the test turns out positive. Thus,

$$\mathbb{E}\left[ FP\left( s \right) \right]=\left( 1-s_{p} \right)P\left( I\left( \mathcal{S} \right)=0 \right)=\left( 1-s_{p} \right)\sum_{n=0}^{N-1} \frac{N-n}{N}q_{R,k,N}\left( n \right).$$

If $\left| \mathcal{S} \right|>1$, a pooled test is performed and, after a positive result, individual tests are performed subsequently. Then, truly negative subjects are falsely classified as positive if the corresponding pooled test outcome is positive and the subject’s subsequent individual test outcome is positive, i.e.,

$$\mathbb{E}\left[ FP\left( s \right) \right]=\sum_{s=0}^{\left| \mathcal{S} \right|-1} P\left( T\left( \mathcal{S} \right)=1 \right| I\left( \mathcal{S} \right)=s)P(I\left( \mathcal{S} \right)=s)(\left| \mathcal{S} \right|-s)(1-s_{p}).$$

Finally, under our assumptions about the individual probabilities, we rewrite the above expression as

$$\mathbb{E}\left[ FP\left( s \right) \right]=\left( 1-s_{p} \right)P\left( I\left( \mathcal{S} \right)=0 \right)\left| \mathcal{S} \right|\left( 1-s_{p} \right)+ \sum_{s=1}^{\left| \mathcal{S} \right|-1} \left( 1-s_{p}+\left( s_{e}+s_{p}-1 \right)\left( \frac{s}{\left| \mathcal{S} \right|} \right)^{d} \right)P\left( I\left( \mathcal{S} \right)=s \right)\left( \left| \mathcal{S} \right|-s \right)\left( 1-s_{p} \right)$$

$$=\left( 1-s_{p} \right)^{2}\left| \mathcal{S} \right|\left[ \sum_{n=0}^{N} \frac{\binom{N-n}{\left| \mathcal{S} \right|}}{\binom{N}{\left| \mathcal{S} \right|}}q_{R,k,N}\left( n \right) \right]+ \sum_{s=1}^{\left| \mathcal{S} \right|-1} \left( 1-s_{p}+\left( s_{e}+s_{p}-1 \right)\left( \frac{s}{\left| \mathcal{S} \right|} \right)^{d} \right)(\left| \mathcal{S} \right|-s)(1-s_{p})\left[ \sum_{n=s}^{N} \frac{\binom{n}{s}\binom{N-n}{\left| \mathcal{S} \right|-s}}{\binom{N}{\left| \mathcal{S} \right|}}q_{R,k,N}(n) \right].$$
