# Supplementary material for: Pooled testing of traced contacts under superspreading dynamics
Source: PLoS Comput Biol. 2022 Mar 28;18(3):e1010008. doi: 10.1371/journal.pcbi.1010008 (PMC8989305; doi:10.1371/journal.pcbi.1010008)
Supplement: S2 Appendix — (DOCX) [file pcbi.1010008.s018.docx]

**S2 Appendix. Dynamic programming algorithm**

Algorithm 1 provides a pseudocode of the dynamic programming algorithm used to determine the optimal pool sizes. Within the algorithm, the function ComputeObjective(·) precomputes the function $g\left( i \right)\mathbb{=E}\left[ K\left( s \right) \right]+\lambda_{1}\mathbb{E}\left[ FN\left( s \right) \right]+\lambda_{2}\mathbb{E[}FP\left( s \right)]$, for each $i\in\{1, \ldots, N\}$, using the formulas derived in S1 Appendix.

**Algorithm 1** It finds the sizes of the optimal set of pools under overdispersion of infected contacts

**Input:** Number of secondary contacts N, sensitivity s_e_, specificity s_p_, parameters R, k, $\lambda_{1}$ and $\lambda_{2}$

1: $\mathcal{S}_{0}\leftarrow\emptyset$

2: $h\left( 0 \right)\leftarrow0$

3: **for** $i\in\{1, \ldots, N\}$ **do**

4: $g\left( i \right)\leftarrow ComputeObjective(i, N, s_{e}, s_{p}, R, k, \lambda_{1},\lambda_{2})$

5: **for** $n\in\{1, \ldots, N\}$ **do**

6: $h\left( n \right) \leftarrow\min_{1\leq j\leq n} [g\left( j \right)+h(n-j)]$

7: $s \leftarrow\underset{1\leq j\leq n}{\mathrm{argmin}} [g\left( j \right)+h(n-j)]$

8: $\mathcal{S}_{n}\leftarrow\mathcal{S}_{n-s}\cup\{s\}$

9: **return** $\mathcal{S}_{N}$

To find the optimal pool sizes given by Dorfman’s method, we use a variation of Algorithm 1, where line 4 is replaced by $g\left( i \right)\leftarrow ComputeObjective(i, N, s_{e}, s_{p}, p, \lambda_{1},\lambda_{2})$. Here, the function ComputeObjective(·) precomputes the function g(i), for each $i\in\{1, \ldots, N\}$, using the formulas derived in S3 Appendix, where p is an independent probability of infection for each contact.

**Proposition 1.** Given N contacts, the set of pool sizes $\mathcal{S}_{N}$ returned by Algorithm 1 are optimal.

**Proof.** We prove this proposition by induction. In the base case, where n = 1, it is easy to see that the optimal solution is $\mathcal{S}_{1}^{*}=\{1\}$, i.e., it consists of one pool of size 1 and the minimum of the objective value is OPT_1_ = g(1), while the recursive functions trivially find the optimal solution since h(1) = g(1). For n > 1 contacts, the inductive hypothesis is that the values h(i) and sets S_i_ recovered by Algorithm 1 for all i < n are optimal. Let $\mathcal{S}_{n}^{*}$ and OPT_n_ be the optimal set of pool sizes and the respective value of the objective function for n contacts.

Suppose, for the sake of contradiction, that OPT_n_ < h(n), i.e., the solution computed using the recursive functions for n contacts is suboptimal. Let $\mathcal{S}_{n}^{*}=\{s_{1}^{*}, s_{2}^{*}, \ldots, s_{l}^{*}\}$. Then, we get:

$$\sum_{i=1}^{l} g\left( s_{i}^{*} \right)<g\left( s \right)+h(n-s)\Rightarrow\sum_{i=1}^{l} g(s_{i}^{*})<g\left( s_{1}^{*} \right)+h\left( n-s_{1}^{*} \right)\Rightarrow\sum_{i=2}^{l} g(s_{i}^{*})<{OPT}_{n-s_{1}^{*}},$$

where the first step is based on the fact that $g\left( s \right)+h\left( n-s \right)\leq g(j)+h(n-j)$ for all $j:1\leq j\leq n$ and the second step is based on the inductive hypothesis. Since $\sum_{i=2}^{l} s_{i}^{*}=n-s_{1}^{*}$, the final inequality implies that, having $n-s_{1}^{*}$ contacts, the set of pool sizes $\left\{ s_{2}^{*}, \ldots, s_{l}^{*} \right\}$ is strictly better than the optimal one which is clearly a contradiction. Therefore, the values h(n) and sets of

pool sizes $\mathcal{S}_{n}$ given by the recursive functions are optimal for all $n:1\leq n\leq N$.
