# Supplementary material for: Pooled testing of traced contacts under superspreading dynamics
Source: PLoS Comput Biol. 2022 Mar 28;18(3):e1010008. doi: 10.1371/journal.pcbi.1010008 (PMC8989305; doi:10.1371/journal.pcbi.1010008)
Supplement: S3 Appendix — (DOCX) [file pcbi.1010008.s019.docx]

**S3 Appendix. Derivations for classic Dorfman’s method (Dorf-Cl)**

**Expected number of tests**

Assuming that each individual has an independent probability of infection p, the expected number of tests $\mathbb{E[}K\left( \mathcal{S} \right)]$ due to a pool $\mathcal{S}$ is:

$$\mathbb{E}\left[ K\left( \mathcal{S} \right) \right]=\left\{ \begin{aligned} 1+f\left( \mathcal{S} \right) \left| \mathcal{S} \right|>1 \\ 1 \left| \mathcal{S} \right|=1 \end{aligned} \right. ,$$

where $f(\mathcal{S)}$ is given by

$$f\left( \mathcal{S} \right)=\left| \mathcal{S} \right|\left[ 1-P\left( T\left( \mathcal{S} \right)=0 \right) \right]$$

$$=\left| \mathcal{S} \right|\left[ 1-\sum_{s=1}^{\left| \mathcal{S} \right|} P\left( T\left( \mathcal{S} \right)=0 \right| I\left( \mathcal{S} \right)=s)P(I\left( \mathcal{S} \right)=s)-P\left( T\left( \mathcal{S} \right)=0 \right| I\left( \mathcal{S} \right)=0)P(I\left( \mathcal{S} \right)=0) \right]$$

$$=\left| \mathcal{S} \right|\left[ 1-\sum_{s=1}^{\left| \mathcal{S} \right|} \left( s_{p}- \left( s_{e}+s_{p}-1 \right)\left( \frac{s}{\left| \mathcal{S} \right|} \right)^{d} \right)\binom{\left| \mathcal{S} \right|}{s}p^{s}\left( 1-p \right)^{\left| \mathcal{S} \right|-s}-s_{p}{(1-p)}^{\mathcal{|S|}} \right],$$

where the last step follows from the fact that $I\left( \mathcal{S} \right) \sim Binom(|\mathcal{S|,}p)$ and our assumptions about $T(\mathcal{S)}$.

**Expected number of false negatives**

Similarly to S1 Appendix, to compute the number of false negatives, we distinguish between two cases. If $\left| \mathcal{S} \right|=1$, a false negative can occur only if the person is infected and the test turns out negative. Thus,

$$\mathbb{E}\left[ FN\left( \mathcal{S} \right) \right]=\left( 1-s_{e} \right)P\left( I\left( \mathcal{S} \right)=1 \right)=\left( 1-s_{e} \right)p.$$

If $\left| \mathcal{S} \right|>1$, a pool test is performed and, if it turns out positive, individual tests are performed subsequently. Then, the expected number of false negatives is

$$\mathbb{E}\left[ FN\left( s \right) \right]=\sum_{s=1}^{\left| \mathcal{S} \right|} sP\left( T\left( \mathcal{S} \right)=0 \right| I\left( \mathcal{S} \right)=s)P\left( I\left( \mathcal{S} \right)=s \right)+\sum_{s=1}^{\left| \mathcal{S} \right|} P\left( T\left( \mathcal{S} \right)=1 \right| I\left( \mathcal{S} \right)=s)P\left( I\left( \mathcal{S} \right)=s \right)s(1-s_{e})$$

$$=\sum_{s=1}^{\left| \mathcal{S} \right|} s\left( s_{p}- \left( s_{e}+s_{p}-1 \right)\left( \frac{s}{\left| \mathcal{S} \right|} \right)^{d} \right)\binom{\left| \mathcal{S} \right|}{s}p^{s}\left( 1-p \right)^{\left| \mathcal{S} \right|-s}+\sum_{s=1}^{\left| \mathcal{S} \right|} s\left( 1-s_{e} \right)\left( 1-s_{p}+\left( s_{e}+s_{p}-1 \right)\left( \frac{s}{\left| \mathcal{S} \right|} \right)^{d} \right)\binom{\left| \mathcal{S} \right|}{s}p^{s}\left( 1-p \right)^{\left| \mathcal{S} \right|-s}$$

$$=\sum_{s=1}^{\mathcal{|S|}} s\left[ 1-s_{e}+s_{e}\left( s_{p}- \left( s_{e}+s_{p}-1 \right)\left( \frac{s}{\left| \mathcal{S} \right|} \right)^{d} \right) \right]\binom{\left| \mathcal{S} \right|}{s}p^{s}\left( 1-p \right)^{\left| \mathcal{S} \right|-s}.$$

**Expected number of false positives**

Similarly, as with the expected number of false negatives, we distinguish between the two cases. If $\left| \mathcal{S} \right|=1$, there is no distinction between a pool test and an individual test. Therefore, a false positive can occur only if the person is not infected and the test turns out positive. Thus,

$$\mathbb{E}\left[ FP\left( s \right) \right]=\left( 1-s_{p} \right)P\left( I\left( \mathcal{S} \right)=0 \right)=\left( 1-s_{p} \right)(1-p).$$

If $\left| \mathcal{S} \right|>1$, a pool test is performed and, after a positive result, individual tests are performed subsequently. Then, truly negative subjects are falsely classified as positive if the corresponding pool test outcome is positive and the subject’s subsequent individual test outcome is positive, i.e.,

$$\mathbb{E}\left[ FP\left( s \right) \right]=\sum_{s=0}^{\left| \mathcal{S} \right|-1} P\left( T\left( \mathcal{S} \right)=1 \right| I\left( \mathcal{S} \right)=s)P(I\left( \mathcal{S} \right)=s)(\left| \mathcal{S} \right|-s)(1-s_{p})$$

$$=\left( 1-s_{p} \right)P\left( I\left( \mathcal{S} \right)=0 \right)\left| \mathcal{S} \right|\left( 1-s_{p} \right)+ \sum_{s=1}^{\left| \mathcal{S} \right|-1} \left( 1-s_{p}+\left( s_{e}+s_{p}-1 \right)\left( \frac{s}{\left| \mathcal{S} \right|} \right)^{d} \right)P\left( I\left( \mathcal{S} \right)=s \right)\left( \left| \mathcal{S} \right|-s \right)\left( 1-s_{p} \right)$$

$$=\left( 1-s_{p} \right)^{2}\left| \mathcal{S} \right|{(1-p)}^{\mathcal{|S|}}+ \sum_{s=1}^{\left| \mathcal{S} \right|-1} \left( 1-s_{p}+\left( s_{e}+s_{p}-1 \right)\left( \frac{s}{\left| \mathcal{S} \right|} \right)^{d} \right)(\left| \mathcal{S} \right|-s)(1-s_{p})\binom{\left| \mathcal{S} \right|}{s}p^{s}\left( 1-p \right)^{\left| \mathcal{S} \right|-s}.$$
