# Supplementary material for: Pooled testing of traced contacts under superspreading dynamics
Source: PLoS Comput Biol. 2022 Mar 28;18(3):e1010008. doi: 10.1371/journal.pcbi.1010008 (PMC8989305; doi:10.1371/journal.pcbi.1010008)
Supplement: Attachment — Submitted filename: PCOMPBIOL-D-21-01826_Review.pdf [file pcbi.1010008.s020.pdf]

Paper: Pool Testing of Traced Contacts Under Superspreading Dynamics (PCOMPBIOL-D-21-01826)

Authors: Stratis Tsirtsis, Abir De, Lars Lorch, Manuel Gomez-Rodriguez

Journal: PLOS Computational Biology

Date: November 10, 2021

Summary:

The paper studies the problem of screening all contacts of a COVID-19 diagnosed individual in order to accurately classify them as positive or negative. The authors achieve this by considering pool testing schemes in which multiple subjects are placed into a pool and tested simultaneously with a single test. One of the simplest pool testing schemes (which is the focus of this paper) was first developed by Dorfman in 1943 and is comprised of two-stages: In the first stage, subjects are placed in a pool which is then tested. If the pool test outcome is negative, then testing stops and all subjects are classified as negative. If the pool test outcome is positive, then every subject in the pool is individually tested and classified according to their individual test outcome. Pool testing can substantially reduce screening costs, especially in settings with low prevalence rates. Owing to such benefits, pool testing has been successfully adopted by many countries to efficiently screen for COVID-19.

The main insight of the paper is that the number of secondary infections caused by an infected individual is overdispersed (i.e., has a high variance). Existing approaches model the number of additional infections through a Poisson distribution whose variance cannot be calibrated and is equal to the mean. The authors propose to overcome this limitation by modeling the number of secondary infections by a truncated generalized negative binomial distribution. The additional parameter of the distribution allows for the calibration of the variance which can be used to model overdispersion. The authors construct a dynamic programming procedure to determine the sequence of optimal pool sizes that minimize an objective function comprised of the expected number of tests and two penalty terms related to the number of false negatives and false positives. They then conduct a series of simulation experiments that demonstrate the benefits of the proposed approach over conventional methods.

Overall, the concept is interesting, the topic is timely, and the paper is well-written. The presentation of ideas is clear, concise, and straight to the point. Moreover, to the best of my knowledge, I believe the modeling of overdispersion within this context is novel. Having said this, I have a number of issues with the paper, mostly related to some assumptions, modeling choices, and the numerical experiments (please see detailed comments below). Considering so, I recommend a **major revision** in which the authors address the below concerns.

Comments

1. What is the motivation for using the truncated generalized negative binomial distribution? Yes, the distribution provides an additional parameter which allows for calibration. However, countless other distributions are also available. Given that the existing body of work assumes a Poisson distribution, have you considered a generalized Poisson distribution, e.g., a mixture of two Poisson distributions? This seems to be a more natural extension.
2. The reproductive number, r, and prevalence rate, p, are often known beforehand. As such, by using these two values one can obtain the overdispersion parameter, k, through the following

formula $k = r(1/p - 1)$. Consequently, while the chosen distribution results in a higher variance over the Poisson distribution (which arises directly by matching the mean of the proposed distribution to r), the proposed model does not allow the modeler to control the variance (as it is hard coded). Isn't this a disadvantage? What if the resulting variance is too low or too high? It seems that the literature provides a more general distribution for the "generalized negative binomial distribution" with additional parameters that can be used to calibrate the variance (in addition to the mean) [1]. Why not utilize this more general distribution?

3. I have some concerns when matching the mean of the distribution to the reproductive number, r. I believe that the final distribution being utilized in the paper is the truncated generalized negative binomial distribution. However, on Page 7 the authors match the mean to the non-truncated distribution. Why is this the case? It seems to me that the mean of the distribution defined in Eq. (1) should be matched to r.

4. The authors make a clear distinction between analytic and clinical test efficacy values. They propose to use analytic sensitivity and specificity data to estimate the dilution parameter value. Then, the authors use this calibrated dilution parameter value to characterize the dilution behavior of clinical sensitivity and specificity data. Based on my knowledge, the existing body of work primarily base their results on analytic sensitivity and specificity data. Given this, I am curious to see how the proposed model compares to the classical Dorfman method when the analytic sensitivity and specificity data are used. Figure 3 shows how the performance measures vary with s_e and s_p, but it does not show how the proposed method compares to the classical Dorfman method.

5. I have some concerns with some of the comparisons performed in the results section. In particular, in Table 1 the authors compare the performance of the proposed method to the classical Dorfman method (through the expected number of tests, false negatives, and false positives) for different values of the number of contacts, N. However, I do not believe that this comparison is necessarily fair. The authors argue that the setting of overdispersion is more realistic, and hence their model is more realistic than the classical Dorfman method. As such, a fairer comparison is as follows: Use the proposed method and the classical Dorfman method to identify the optimal pool sizes for each method. Then, by using the expressions of the proposed method only (because it is a better reflection of reality), evaluate the difference in performance for the two pool sizes. That is, had one based their answer on the classical Dorfman method, the observed performance should be measured through the more realistic approach that incorporates overdispersion. Doing so quantifies the true difference in performance. This change also impacts other components of the results section (e.g., Figure 1(B)).

6. In Algorithm 1 in the appendix, the caption uses k as an index while the main algorithm uses i as an index. For consistency, I would recommend having both set to i.

7. I believe there is a a missing comma on Line 423 right after $h(n)$.

References

[1] GC Jain and PC Consul. A generalized negative binomial distribution. *SIAM Journal on Applied Mathematics*, 21(4):501–513, 1971.
