# Supplementary material for: Pooled testing of traced contacts under superspreading dynamics
Source: PLoS Comput Biol. 2022 Mar 28;18(3):e1010008. doi: 10.1371/journal.pcbi.1010008 (PMC8989305; doi:10.1371/journal.pcbi.1010008)
Supplement: Attachment — Submitted filename: PLOS revision response.pdf [file pcbi.1010008.s021.pdf]

# Response Letter:
# “Pooled Testing of Traced Contacts Under Superspreading Dynamics”
# (PCOMPBIOL-D-21-01826)

Stratis Tsirtsis, Abir De, Lars Lorch and Manuel Gomez-Rodriguez

We thank all reviewers for their thoughtful comments and feedback. We have revised our manuscript to address all the reservations, comments and feedback shared by the reviewers. These were mainly related to the motivation of our work and its modeling assumptions as well as implementation details of our experimental design. In this response letter, we first reply to two points raised by both Reviewers 2 and 3. Then, we reply to each of the remaining points raised by each reviewer individually.

***On the motivation to use the truncated generalized negative binomial distribution***

We agree with the reviewers that one could consider alternative distributions to characterize the number of secondary infections caused by an infectious individual. Although this is an interesting area of research, with mixture models being a natural candidate [1], we decided to use the generalized negative binomial distribution because it is the most popular choice in the literature studying superspreading phenomena of infectious diseases [2-7]. In lines 69-70 of the revised manuscript, we explicitly motivate our choice and point the reader to related prior work.

In addition, please note that the probability mass function of the truncated generalized negative binomial distribution can be completely characterized by specifying the values of the reproductive number R and the dispersion parameter k. Here, the modeler can increase (decrease) the variance by decreasing (increasing) the value of k, as noted in lines 96-100 of the revised manuscript. Estimates of both R and k using real contact tracing data are readily available in the recent literature on COVID-19 [4-6]. In this context, we would also like to clarify that we rely on these estimates of R and k to parameterize the generalized negative binomial distribution–-we do *not* make use of the prevalence rate, as Reviewer 2 suggests. The parameter $p = R / (k + R)$, initially introduced in lines 90-92, has no physical interpretation in the context of epidemiology and is not to be confused with the prevalence rate.

***On the implementation of Dorfman’s method***

To compare our method with Dorfman’s, we first generate infection statuses for a set of N contacts such that the number of infections follows an overdispersed truncated generalized negative binomial distribution. Then, we compute the optimal pool sizes given by our method and Dorfman’s using the dynamic programming algorithm (S2 Appendix), with the only difference being in the expressions for the expected numbers of tests, false negatives and false positives that the algorithm uses for either method. Specifically, the ones used by our method (S1 Appendix) account for overdispersion, while the ones used by Dorfman’s method (S3 Appendix) assume an independent probability of infection for each individual contact. Finally, based on the respective pool sizes, we evaluate each method in terms of its average numbers

of resulting tests, false negatives and false positives on the generated set of contacts. Therefore, we believe that our evaluation process is in line with what Reviewer 2 suggests (*"Use the proposed method and the classical Dorfman method to identify the optimal pool sizes for each method. Then, by using the expressions of the proposed method only (because it is a better reflection of reality), evaluate the difference in performance for the two pool sizes."*). In the revised manuscript, we clarify how the evaluation is performed and how the two methods differ in terms of implementation in lines 170-180 of the revised manuscript and 21-24 of S2 Appendix.

## **Reviewer 1**

*"My major concern with this work is that the impact on the actual epidemic is not investigated. If I understand it correctly, temporal (e.g. viral load progression) and contact-related aspects of the epidemic are all aggregated in the clinical sensitivity/specificity (line 170). However, missing contacts, especially in an overdispersed epidemic context, could have implications on the overall attack rate. The authors mention (in the discussion), that this could be investigated through randomized control studies, yet I would argue that this could also be analysed by using an individual-based model to investigate the impact of your testing strategy? To this end, perhaps one of these individual-based models could be used [2,3,4]?"*

We would like to highlight that the goal of our paper is *not* to investigate the effect of pooled testing on the evolution of an ongoing epidemic (e.g., via the attack rate). Instead, our goal is to show that our method can achieve fewer tests in comparison to Dorfman's by accounting for the overdispersion of secondary infections. We mention this goal explicitly in the Abstract (lines 14-17), the Introduction (lines 76-78) and the Experimental Design (lines 166-168) of our revised manuscript and we empirically show that our method achieves this goal in Results (lines 204-212, 238-246). While we acknowledge that imperfect contact tracing can have a negative impact on the containment of an epidemic, we also show that our method maintains its competitive advantage with respect to Dorfman's even when a significant number of contacts is unreported (lines 316-325). To avoid misunderstandings, in lines 355-356 of the Discussion, we clarify the type of randomized control study that we consider an interesting direction for future work.

That being said, we agree with the reviewer that it would be very interesting to investigate the impact of different testing methods on the epidemic under a limited testing capacity using individual-based models. However, this would require considering a large number of additional parameters such as, e.g., the number of available daily tests, the exact quarantine regulations for positively diagnosed individuals, the contact tracing strategy or the lab tests' turnaround time, to name a few. As a result, we believe such an investigation goes beyond the scope of our work.

*"Did you consider any additional population structures next to the contact tracing contacts (e.g., households), and how would this fit in your work? Adjacent to this, the related work section was quite complete, however I believe that the work on household-based pooling [1] could also be interesting to discuss."*

We thank the reviewer for the interesting reference. In lines 161-164 of our revised manuscript, we explain how our method can be used to assign a fraction of the contacts into pools, while assigning the remaining contacts manually (e.g., based on their membership to a common household), and cite the above reference.

*"I found Figure 1 (b) a bit strange and hard to interpret at first sight. It looks strange with the negative percentages, could you perhaps show the test distributions for both methods instead next to each other?"*

Note that the numbers of tests performed by both methods present high variance, as explicitly noted in lines 212-213, and they depend not only on the pool sizes computed by each method but also on the number of infections in each generated set of contacts. The goal of Figure 1(b) is to show, for each generated set of contacts, what percentage of the tests performed by Dorfman's method would have been saved, had we used our method instead. We consider this information to be more insightful than the one gained by a side-by-side comparison of the test distributions of the two methods and this is the reason why we opted for this particular type of plot. Although the mean of the presented (empirical) distribution is clearly positive (our method would have saved a significant amount of tests on average), the negative values of the distribution have also a natural interpretation. For example, a percentage of -100% means that our method would have performed double the amount of tests in comparison to Dorfman's.

## Reviewer 2

*"I have some concerns when matching the mean of the distribution to the reproductive number, r. I believe that the final distribution being utilized in the paper is the truncated generalized negative binomial distribution. However, on Page 7 the authors match the mean to the non-truncated distribution. Why is this the case? It seems to me that the mean of the distribution defined in Eq. (1) should be matched to r."*

We would like to thank the reviewer for this thoughtful question. In lines 88-97 of the revised manuscript, we explain how we parameterize the generalized negative binomial distribution via the reproductive number R and the dispersion parameter k. Note that the reproductive number R reflects the average number of secondary infections caused by an arbitrary individual in the population (with an unknown number of contacts). This is the reason for matching the mean of the aforementioned *non-truncated* distribution to the reproductive number R (line 97). Following from that, the number of infections caused by an individual with N contacts is upper-bounded by N and follows a *truncated* generalized negative binomial distribution, as described in lines 101-105.

*"The authors make a clear distinction between analytic and clinical test efficacy values. They propose to use analytic sensitivity and specificity data to estimate the dilution parameter value. Then, the authors use this calibrated dilution parameter value to characterize the dilution behavior of clinical sensitivity and specificity data. Based on my knowledge, the existing body of work primarily base their results on analytic sensitivity and specificity data. Given this, I am curious to see how the proposed model compares to the classical Dorfman method when the analytic sensitivity and specificity data are used. Figure 3 shows how the performance*

*measures vary with se and sp, but it does not show how the proposed method compares to the classical Dorfman method."*

In this work, as we argue in lines 181-187 of the revised manuscript, we mainly focus on clinical sensitivity and specificity values since our experimental setup emulates pooled testing of samples collected through contact tracing. However, in the supporting information, we provide results where we compare our method to Dorfman's (similar to the results in Table 1 and Fig. 1) under additional values of sensitivity and specificity, including the (analytic) values we used for the estimation of the dilution parameter (S3 Table, S2 Fig.).

## Reviewer 3

*"General comment on the case study. Since you are interested in minimizing a weighted sum of expected number of tests, false-negatives, and false-positives, then why don't you report the value of that objective function in your comparison of your approach with the Dorfman scheme?"*

We would like to clarify that we do compare the value of the objective function achieved by our method and Dorfman's in Figure 4(A) and Tables 1, S1, S2 and S3. However, in those experiments, we set $\lambda_1=\lambda_2=0$ and, therefore, the value of the objective function is equal to the average number of tests that we report in the respective Figure/Tables.

*"Would it be possible to run your method by only accounting for false negatives? It would be interesting to compare your approach to Dorfman testing if the only goal is to minimize negative misclassification errors."*

We would like to point out that, if the only goal is to minimize negative misclassification errors ($\lambda_1 \rightarrow \infty$), the optimal pool sizes given by both methods correspond to individual testing and, therefore, the two methods become equivalent. In this context, we would also like to point out that, when $\lambda_2 \rightarrow \infty$, both methods also become equivalent and choose pools of size two. We include this clarification in lines 279-280 of the revised manuscript.

## References


[1] Kremer, C., Torneri, A., Boesmans, S. et al. "Quantifying superspreading for COVID-19 using Poisson mixture distributions." Sci Rep 11, 14107 (2021).

[2] Lloyd-Smith, James O. "Maximum likelihood estimation of the negative binomial dispersion parameter for highly overdispersed data, with applications to infectious diseases." PloS one 2.2 (2007): e180.

[3] Lloyd-Smith, James O., et al. "Superspreading and the effect of individual variation on disease emergence." Nature 438.7066 (2005): 355-359.

[4] Adam, Dillon C., et al. "Clustering and superspreading potential of SARS-CoV-2 infections in Hong Kong." Nature Medicine 26.11 (2020): 1714-1719.

[5] Endo, Akira. "Estimating the overdispersion in COVID-19 transmission using outbreak sizes outside China." Wellcome open research 5 (2020).

[6] Lau, Max SY, et al. "Characterizing superspreading events and age-specific infectiousness of SARS-CoV-2 transmission in Georgia, USA." Proceedings of the National Academy of Sciences 117.36 (2020): 22430-22435.

[7] Kucharski, A. J., and Christian L. Althaus. "The role of superspreading in Middle East respiratory syndrome coronavirus (MERS-CoV) transmission." Eurosurveillance 20.25 (2015): 21167.
