# Supplementary material for: Pooled testing of traced contacts under superspreading dynamics
Source: PLoS Comput Biol. 2022 Mar 28;18(3):e1010008. doi: 10.1371/journal.pcbi.1010008 (PMC8989305; doi:10.1371/journal.pcbi.1010008)
Supplement: Attachment — Submitted filename: PLOS revision response 2.pdf [file pcbi.1010008.s022.pdf]

Response Letter:
“Pooled Testing of Traced Contacts Under Superspreading Dynamics”
(PCOMPBIOL-D-21-01826R1)

Stratis Tsirtsis, Abir De, Lars Lorch and Manuel Gomez-Rodriguez

We thank all reviewers and editors for carefully reading our response to the manuscript’s initial reviews and the revised manuscript. We have revised our manuscript to address one last comment by Reviewer 1, related to the discussion of future work.

Reviewer 1

I thank the authors for their answers and improvements of the manuscript. One more remark: on my comment regarding the impact on the testing strategy on the epidemic, the authors state: "That being said, we agree with the reviewer that it would be very interesting to investigate the impact of different testing methods on the epidemic under a limited testing capacity using individual-based models." It would be good to indeed mention this in the discussion.

In lines 354-356 of our revised manuscript, we comment on the potential of using individual-based models to study the effect of different testing methods on the evolution of an epidemic under limited testing resources. As examples of such models, we cite the relevant papers suggested by the reviewer in their original review [1, 2, 3].

References

[1] Kerr, Cliff C., et al. "Covasim: an agent-based model of COVID-19 dynamics and interventions." PLOS Computational Biology 17.7 (2021): e1009149.

[2] Hinch, Robert, et al. "OpenABM-Covid19—An agent-based model for non-pharmaceutical interventions against COVID-19 including contact tracing." PLOS Computational Biology 17.7 (2021): e1009146.

[3] Willem, Lander, et al. "The impact of contact tracing and household bubbles on deconfinement strategies for COVID-19." Nature communications 12.1 (2021): 1-9.
